# Standardized Rapid Sequence Intubation (RSI) Improves Effectiveness and Safety in Mixed Physician and Paramedic Hungarian EMS

**DOI:** 10.3390/life15111725

**Published:** 2025-11-07

**Authors:** Béla Burány, Péter Temesvári, Márton Radnai, Ákos Sóti, Gábor Csukly, Gábor Élő

**Affiliations:** 1 School of PhD Studies, Semmelweis University, 1094 Budapest, Hungary; 2 Emergency Medical Centre, North-Pest Central Hospital—Military Hospital, 1097 Budapest, Hungary; petertemesv@gmail.com; 3 Hungarian Air Ambulance Nonprofit Ltd., 2040 Budaörs, Hungary; radnai.marci@gmail.com (M.R.); soti.akos.tamas@semmelweis.hu (Á.S.); 4 National Ambulance Service, 1087 Budapest, Hungary; 5 Department of Emergency Medicine, Semmelweis University, 1094 Budapest, Hungary; 6 Department of Psychiatry and Psychotherapy, Semmelweis University, 1094 Budapest, Hungary; sugab@yahoo.com; 7 Department of Anaesthesiology and Intensive Therapy, Semmelweis University, 1094 Budapest, Hungary; elo.gabor@semmelweis.hu

**Keywords:** intubation, RSI, EMS, paramedic, DASH-1A, success

## Abstract

(1) Background: Ground Emergency Medical Services in Hungary are provided by the National Ambulance Service. Paramedics, physicians, and specialists in this service are competent in performing endotracheal intubation (ETI) on patients. The aim of this study is to evaluate the impact of the standardized Rapid Sequence Intubation (RSI) procedure on safety and effectiveness. (2) Methods: A retrospective observational study was conducted concerning the RSI procedure. Patient documentation from a 2-year implementation period was analyzed using a dedicated Case Report Form (CRF), where both RSI and non-RSI methods were used. Our primary endpoint was Definitive Airway Sans Hypoxia and Hypotension on First Attempt (DASH-1A). Our secondary endpoints included success on the first attempt; overall success; and hypoxia, hypotension, and cardiac arrest complications. (3) Results: In total, 6399 intubation cases were studied; non-RSI was used in 3236, and RSI was applied in 3163 cases. DASH-1A was attained in a significantly higher number of cases with RSI than non-RSI (55.0 vs. 68.5%, *p* < 0.0001). The DASH-1A results of the RSI group were significantly better in the paramedic (54.0 vs. 68.5%, *p* < 0.0001) and the physician (55.0 vs. 66.7%, *p* = 0.0017) subgroups. In the specialist subgroup, the difference was not statistically significant (64.5 vs. 69.7%, *p* = 0.1514). (4) Conclusions: Standardized RSI significantly increased effectiveness and safety in the paramedic and physician subgroups.

## 1. Introduction

Prehospital endotracheal intubation (ETI) remains a debated topic [1,2,3,4,5,6,7,8,9,10,11,12,13,14,15,16,17,18,19,20,21,22,23], and drug-assisted ETI by paramedics is even more controversial [5,9,10,11,12,13,14,15,24,25,26,27,28,29,30]. Despite conflicting evidence, drug-assisted ETI has remained a routine practice of many pre-hospital services for decades.

Most experts will agree that good clinical governance behind this procedure is essential for its success and safety [2,2,10,13,14,29,31,32,33,34,35,36,37]. We found very little evidence on paramedic drug-assisted ETI from continental Europe [26,38,39].

Emergency Medical Services (EMS) in Hungary are organized and funded by the state, and ground EMS is provided nationwide by the National Ambulance Service (NAS). The service employs around 7500 individuals and responds to more than 1.1 million calls per year. The NAS employs close to 800 paramedics and 300 physicians to provide advanced pre-hospital care. These clinicians have been competent in drug-assisted intubation since the 1970s.

Paramedics in Hungary receive 4 years of college-level training before they receive their diploma, endowing them with almost the same competence level as pre-hospital physicians. Moreover, drug-assisted ETI is part of their training. Physicians working for the NAS are primarily emergency medicine trainees, but trainees of other specialties are also employed, with many on a part-time basis. Specialist doctors within the NAS are considered specialists if they have successfully completed their training in emergency medicine or anaesthesia and intensive care. While these providers traditionally had the highest competence levels, the differences between provider groups have diminished in recent decades. ETI competences are identical and legally equivalent to the three groups of providers (specialists, trainee doctors, and paramedics); however, before 2013, none of the provider subgroups had a Standard Operating Procedure (SOP) to follow. Furthermore, before the introduction of the standardized method, no paralytic medication was available in the prehospital setting in Hungary.

In 2013, a repeated audit observed significant problems regarding the safety of drug-assisted ETI within the service.

Following the international benchmark, a decision was made by management to implement a clinical governance model for Rapid Sequence Intubation (RSI) adapted from Helicopter Emergency Medical Services in the United Kingdom [40,41]. This model was previously successfully implemented by a major emergency department in Hungary (Military Hospital in Budapest) and by the Hungarian Air Ambulance [32]. The SOP identifies the following indications for RSI: prolonged unconsciousness, potential or real airway compromise, ventilatory failure, cerebral agitation due to head injury, and “other” indications; all these are based on a careful risk–benefit analysis. Other elements of the SOP included standardized preoxygenation; patient and operator positions; equipment preparation and confirmation with a challenge–response checklist; standardized induction agents and a short-acting paralytic; failed laryngoscopy and failed intubation plans; and post-intubation care (for details, see Supplementary Digital Content/SDC/Figure S1).

Clinical governance elements included a proven and simple to follow SOP, two-day training, on-call telephone advice by senior specialists, and continuous audits and feedback. Simultaneously, equipment and additional drugs for RSI were made standard equipment on every advanced-level ambulance and response car. Training for the new method started in August 2013, and the transition period took almost two years, during which time the previous, non-standardized method of ETI without muscle relaxants was performed in parallel to the newly implemented RSI procedure. The choice between using the two methods was not carried out by the preference of the provider, as they were expected to follow the RSI SOP after training and equipment criteria were met.

According to our hypothesis, structured RSI training enhances the safety and quality of prehospital ETI. Our study aims to identify the effect (direction and extent) of RSI implementation on the effectiveness and safety of ETI compared to the previous practice of the NAS.

## 2. Materials and Methods

A retrospective cohort study was conducted using a dedicated report form (RF) on previously obtained structured patient documentation in the period 1 August 2025–30 September 2025. A prospective data collection of routine patient documentation was conducted to study ETI within the NAS (trial registration: 244/2025 SE RKEB, Semmelweis University). The study period of the collected structured patient documentation was between 19 September 2013 and 19 September 2015, covering 2 years from the first performed RSI procedure within the NAS.

During these 24 months, all cases where patients were intubated while maintaining spontaneous cardiac activity were studied. We excluded all ETI cases performed on cardiac arrest. Data on the patient population, providers, indications, the ETI method used, success rates, and complications were collected. There was no need for sample size calculation, as the study size was equal to the entire studied population, simultaneously decreasing sample size bias.

Two groups of drug-assisted ETI cases were identified. The first group, termed the RSI group, consisted of providers who followed the RSI SOP after receiving training. This group used an induction muscle relaxant (suxamethonium) and an induction agent (ketamine or etomidate). The other group, termed the non-RSI group, consisted of providers who applied their clinical judgement to identify indications for drug-assisted ETI and to select the methods and drugs for the procedure. This group did not use muscle relaxants.

Provider subgroups were formed for specialists, physicians, and paramedics.

Our data source comprised complete patient documentation that included a printout of monitor trends, where available. Our data went through a validation process, where ten experts in prehospital ETI examined all data and corrected any identified documentation discrepancies—using the available objective data—where possible. We consider end tidal capnography (etCO_2_) as the strongest indicator of a successful definitive airway [42,43,44,45,46,47].

Procedure-related complications were identified as a 10% decrease or a decrease to below 90% in oxygen saturation, a 30% decrease or a drop below 90 mmHg in systolic blood pressure, and cardiac arrest (CA), if any of these complications occurred within 5 min of induction and an intubation attempt. Our primary endpoint was Definitive Airway Sans Hypoxia and Hypotension on First Attempt (DASH-1A) and ETI success rate of the first attempt without major complications [48,49,50].

One intubation attempt was defined by the insertion of the laryngoscope with the intent to intubate without withdrawal of the blade. A second attempt was defined by the repeated insertion of the blade after withdrawal or laryngoscopy lasting more than 60 s.

The data were retrieved from previously obtained structured patient documentation using a dedicated report form containing all relevant and above-mentioned data. No cases were excluded because of a lack of data.

Statistical analyses were performed using SAS software version 9.4 (SAS Institute Inc., Cary, NC, USA). For the primary endpoint, DASH-1A was viewed as a binary variable, and the score differences between the groups (RSI vs. non-RSI) were investigated via a chi-square test (ChiSq).

The effect of risk factors on DASH-1A (dependent variable) and the interactions between the RSI groups and risk factors (independent variables) were explored via logistic regression analyses (PROC LOGISTIC in SAS). The association between the dependent and independent variables in the logistic regression model was tested using the Wald ChiSq statistic (WChiSq). Effect sizes are provided in terms of odds ratios. In all analyses, the alpha level of significance was set to 0.05, while *p* values between 0.05 and 0.1 were referred to as the statistical trend.

In the primary analysis, the effect of RSI on DASH-1A was investigated in a logistic regression model, where gender, age, complications, provider subgroups, trauma, and indications were included as covariates. In the secondary analysis, the effect of RSI and its interactions with the above-mentioned factors were analyzed in separate models.

## 3. Results

During the study period, a total of 2,207,864 missions were completed by the NAS. The above criteria were met in 6399 cases, and the main characteristics are shown in Table 1.

Of the 6399 cases, nearly half were in the non-RSI group (3236 cases, 50.57%), while the remainder belonged to the RSI group (3163 cases, 49.43%).

The majority of ETIs were performed by paramedics (5023 cases, 78.5%). Physicians and specialists were represented with similar percentages (695 cases, 10.86% and 667 cases, 10.42%). We could not identify the provider training background in 14 cases (0.22%).

The incidences of indications for ETI are shown in Table 2. Providers selected indications from multiple-choice options.

Table 2 also shows the trauma patient rate within each group. Trauma patients were significantly more frequent in the RSI group (non-RSI vs. RSI: 313 vs. 468 cases, 9.7 vs. 14.8%, ChiSq = 39.2, *p* < 0.0001). This is true within the paramedic and physician subgroups, while the specialists’ results exhibit a similar but not significant tendency (paramedics: 238 vs. 342 cases, 9.2 vs. 14.1%, ChiSq = 29.2, *p* < 0.0001; physicians: 36 vs. 64 cases, 10.9 vs. 17.5%, ChiSq = 6.0, *p* = 0.01; specialists: 39 vs. 61 cases, 12.7 vs. 16.9%, ChiSq = 2.3 *p* = 0.13).

Table 3 shows the main effectiveness and complication results of the two ETI methods.

The overall success rate of ETI was 96.75% in the two groups. No difference was found between the two groups in this respect (non-RSI vs. RSI: 3120 vs. 3071 cases, 96.4 vs. 97.1%, ChiSq = 2.3, *p* = 0.13). Tube position was verified with capnography in a significantly higher number of cases within the RSI group compared to the non-RSI group (non-RSI vs. RSI: 2502 vs. 3087 cases, 77.3 vs. 97.6%, ChiSq = 595.0, *p* < 0.0001). This was true within all provider subgroups (paramedics: 1986 vs. 2386 cases, 76.7 vs. 98.1%, ChiSq = 512.1, *p* < 0.0001; physicians: 262 vs. 347 cases, 79.6 vs. 94.8%, ChiSq = 36.8, *p* < 0.0001; specialists: 249 vs. 349 cases, 81.1 vs. 96.9%, ChiSq = 44.8, *p* < 0.0001).

We observed a combined first-attempt success rate of 80.14%. The first-pass success rate was significantly higher in the RSI group compared to the non-RSI group (non-RSI vs. RSI: 2422 vs. 2706 cases, 74.9 vs. 85.6%, ChiSq = 115.2, *p* < 0.0001).

Table 3 shows the main complications, where the hypoxia rate did not differ significantly (non-RSI vs. RSI: 249 vs. 264 cases, 7.7 vs. 8.3%, ChiSq = 0.9, *p* = 0.34). This was observed within the provider subgroups as well (paramedics: 198 vs. 208 cases, 7.6 vs. 8.6%, ChiSq = 1.4, *p* = 0.2366; physicians: 28 vs. 29 cases, 8.5 vs. 7.9%, ChiSq= 0.08, *p* = 0.7782; specialists: 22 vs. 27 cases, 7.2 vs. 7.5%, ChiSq = 0.03, *p* = 0.8692).

Hypotension was observed in a significantly higher number of cases in the non-RSI group (non-RSI vs. RSI: 631 vs. 421 cases, 19.5 vs. 13.3%, ChiSq = 44.6, *p* < 0.0001). This significant difference was found in the paramedic (506 vs. 319 cases, 19.5 vs. 13.1%, ChiSq = 37.6, *p* < 0.0001) and physician subgroups (76 vs. 50 cases, 23.1 vs. 13.7%, ChiSq = 10.4, *p* = 0.0013); in contrast, in the specialist subgroup, the difference was not significant (48 vs. 51 cases, 15.6 vs. 14.2%, ChiSq = 0.28, *p* = 0.5949).

CA as a complication was observed in less than 5% of cases in both groups. The RSI group had slightly—but not significantly—lower CA rates compared to the non-RSI group (non-RSI vs. RSI: 107 vs. 85 cases, 3.3 vs. 2.7%, ChiSq = 2.1, *p* = 0.15). No significant difference was found between the non-RSI and RSI groups within either of the provider subgroups (paramedics: 82 vs. 65 cases, 3.2 vs. 2.7%, ChiSq = 1.07, *p* = 0.3011) (physicians: 12 vs. 13 cases, 3.7 vs. 3.6%, ChiSq = 0.00, *p* = 0.9462) (specialists: 13 vs. 7 cases, 4.2 vs. 1.9%, ChiSq = 2.99, *p* = 0.0839).

The effect of RSI (non-RSI vs. RSI) on DASH-1A was investigated using a Logistic Regression model and Wald ChiSq test (WChiSq), where gender, age, provider subgroup, trauma, and indications were included as covariates. As the primary endpoint, DASH-1A was reached in a significantly higher number of cases in the RSI group compared to the non-RSI group in both the paramedic (RSI vs. non-RSI: OR = 1.86 [95% CI = 1.65–2.08], n = 3066, 68.5 vs. 54.0%) and physician (RSI vs. non-RSI: OR = 1.64 [95% CI = 1.20–2.22], n = 425, 66.7 vs. 55.0%) subgroups. In the specialist subgroup, the RSI results remained superior; however, the difference did not reach statistical significance (RSI vs. non-RSI: OR = 1.27 [95% CI = 0.92–1.75], n = 449, 69.7 vs. 64.5%).

In the non-RSI group, the DASH-1A rates did not differ significantly between the paramedic and physician subgroups (54.0 vs. 55.0%, *p* = 0.73), while the results of the specialist subgroup were significantly better compared to the paramedic (paramedic vs. specialist: 54.0 vs. 64.5%, *p* = 0.0005) and physician subgroups (physician vs. specialist: 55.0 vs. 64.5%, *p* = 0.01).

In the RSI group, the differences between provider subgroups are not significant (paramedic vs. physician: 68.5 vs. 66.7%, *p* = 0.47; physician vs. specialist: 66.7 vs. 69.7%, *p* = 0.38; paramedic vs. specialist: 68.5 vs. 69.7%, *p* = 0.65).

Trauma did not have the main effect on DASH-1A (WChiSq = 1.8, *p* = 0.18), while RSI (WChiSq = 67.5, *p* < 0.0001) and the interaction between RSI and trauma (WChiSq = 3.8, *p* = 0.05) had a significant effect on DASH-1A. For non-trauma patients, the odds of DASH-1A were significantly increased in the RSI group compared to the non-RSI group (WChiSq = 100.3, *p* < 0.0001, OR = 1.75, 95% CI: 1.57–1.95) (Table 4).

For trauma patients, the results in the RSI group were even better, doubling the odds (WChiSq = 23.9, *p* < 0.0001, OR = 2.09, 95% CI: 1.55–2.80). In the RSI group, the DASH-1A odds were similar for trauma and non-trauma patients (WChiSq = 0.1, *p* = 0.7, OR: 0.96, 95% CI: 0.78–1.19), while in the non-RSI group, the odds for trauma patients were worse, but only at a tendency level (WChiSq = 3.3, *p* = 0.07, OR: 0.81, 95% CI: 0.64–1.02) (Table 4). In summary, a history of trauma had no main or modifying effect on the effectiveness of the RSI method.

The interaction effect of RSI and age on DASH-1A was analyzed via a separate logistic regression analysis, with RSI, age, and their interactions as predictor variables. Age did not have a main effect on DASH-1A (WChiSq = 1.7, *p* = 0.19), while RSI (WChiSq = 31.7, *p* < 0.0001) and the interaction between RSI and age (WChiSq = 7.4, *p* = 0.006) had a significant effect on DASH-1A. The post hoc analysis showed that RSI was superior to non-RSI in all age groups, although the odds ratios decreased with age.

There is a major difference between the non-RSI and RSI subgroups regarding the incidence of indications (Table 2). When the effects of indications were analyzed in separate logistic regression models with respect to RSI, the given indication, and their interaction as predictor variables, RSI had a significant main effect in all models (OR ranging between 1.4 and 2.9), while only ventilatory failure (WChiSq = 9.1, *p* = 0.003) and airway compromise (WChiSq = 3.9, *p* = 0.049) had significant interactions with RSI, indicating that these indications affect the odds ratios between RSI and non-RSI (Table 4). The indications did not have a significant main effect on DASH-1A in any of the models (*p* > 0.05).

## 4. Discussion

Based on previous audit results, the following changes were implemented in the pre-hospital care system in Hungary, specifically in the NAS:

• Standardized equipment and training for clinicians;
• Modification of recommended drugs for induction;
• Introduction of muscle relaxants for the induction of intubation;
• After having identified ETI as a critical process, clinical governance elements were introduced (SOP development and updates, simulation-based training, real-time telephone consultation system, continuous feedback and audit, etc.).

The above changes were also based on the demonstrated feasibility of the Hungarian system, as shown by the Hungarian Air Ambulance [32].

All these factors could have contributed to the differences observed between the non-RSI and RSI groups. It remains unclear how these factors influenced the changes in effectiveness measured in this study, in what proportions, and with what possible interactions.

We concur that the gold standard for ET tube position verification is etCO_2_ measurement [42,43,44,45,46,47]. However, in 18% of the cases, etCO_2_ data were unavailable (mainly because of equipment issues) to support the documentation of a definitive airway that had been confirmed by other clinical means. This missing data occurred mostly in the non-RSI group. From another perspective, SOP compliance was better in the RSI group in this sense as well. We examined data without etCO_2_ in more detail. Our analysis showed that the exclusion of cases without etCO_2_ data would only have increased the difference in effectiveness between the RSI and non-RSI groups. Finally, we decided to include data without etCO_2_ values in this study.

The overall success rate of ETI in our database—measured during the study period—is 96.4% in the non-RSI group and 97.1% in the RSI group. This result is acceptable by international standards [10,24,25,32,34,39,40,45,51].

One of the main goals of pre-hospital ETI is to achieve a definitive airway on the first attempt [34,35]. The combined first-attempt success rate across both groups in this NAS setting was markedly lower than that recently published by Helicopter Emergency Medical Services (HEMS): Harris and Lockey reported 87.5% in 2011, and Sunde et al. reported 89% in 2015 and 90% in 2017 with respect to physician-based helicopter services [40,51,52]. In this respect, the Hungarian HEMS result of 95.4% is outstanding [32]. First-attempt success rates in the non-RSI group were sub-standard in the paramedic and physician subgroups compared to international results [24,32,35,39,49,53,54]. With the RSI method, paramedics, physicians, and specialists all achieved success rates approaching the 88% reported by physician-led services and the international Emergency Department benchmark of 84.1% [36,39,55].

In this study, we identified complications (hypoxia, hypotension, and CA) known to be detrimental to patients’ long-term quality of life [40,51,52]. These events were classified as complications if they occurred within 5 min of the start of the intubation procedure.

The hypoxia rate did not significantly differ in the two main groups or between the three subgroups. The rates are slightly higher than those published by other services [32,39,51,52].

Some of the differences may be attributable to variations in the patient population (different rates of respiratory failure and trauma), although differences in the quality of care cannot be excluded.

The hypotension rate was significantly higher in the non-RSI paramedic and physician subgroups. After the implementation of the RSI procedure, all three subgroups exhibited similar results, although the documented occurrence remains higher than the internationally reported occurrences [32,39,51,52,56].

The occurrence of CA did not differ or improve significantly, and again, the rates are marginally above international standards [24,32,52,56].

The combined indicator DASH-1A was used as a primary endpoint [48,49,50]. DASH-1A was 68.5% in the RSI group, which is a major improvement over the 55.0% measured in the non-RSI group. This indicates that the RSI system significantly improved the effectiveness of paramedic and physician ETI. In contrast, with respect to specialists, we observed a difference that was not statistically significant.

The implementation of the RSI system—which was not only a new method but also an extended quality improvement that included equipment development, SOPs, training, inspections, feedback, etc.—significantly increased the first-attempt success rate and reduced the occurrence of hypotension within the paramedic and physician subgroups.

Given the limited number of specialists in the pre-hospital field in Hungary, with 4/5 of all ETIs performed by paramedics and an additional 10% performed by non-specialist physicians, it is crucial that, under the SOP-based RSI system, paramedics and non-specialist physicians achieve performance comparable to that of the specialists. Although the implementation of the new SOP led to internationally comparable results in most areas, we also recognize that there remain aspects in which all provider groups can still improve.

Although our study has several strengths—such as a large case database analyzing the effects of quality improvement changes implemented on a country-wide, comprehensive scale in the pre-hospital system—we must also acknowledge some limitations:

• After training and with equipment available, all providers were required to follow the new SOP; however, occasional cases still employed the non-RSI method.
• The SOP was known to all providers before training; this might have influenced the non-RSI arm.
• The database relied on self-reported data, which inherently carries the risk of documentation bias.
• In addition to the examined factors, the effectiveness of ETI may have been influenced by other variables that were not analyzed in this study (e.g., comorbidity, preoxygenation effectiveness, pre-induction haemodynamic status, etc.); these variables may have differed between the subgroups, even with the large number of cases. These variables could be the subject of future research [35].

## 5. Conclusions

Ground-based EMS in Hungary is provided by a single operator: the NAS. Following Emergency Department and Hungarian HEMS trials, standardized RSI for pre-hospital drug-assisted intubation was introduced widely into the service, and this was supported by a complex clinical governance structure. Using the up-to-date endpoint DASH-1A, our study of the first two years of the new practice demonstrates, on a large case series, that the RSI method (which is proven to be effective in several other countries) improved both effectiveness and safety in a Central European setting. This was true not only when performed by specialist or non-specialist physicians but also by paramedics for both trauma and non-trauma patients, compared with their previous practice, which relied solely on provider training and experience.

The introduction of the SOP-based RSI system not only improved effectiveness and safety but also improved equality of care in Hungarian pre-hospital practice.

## Figures and Tables

**Table 1:** Demographic data.

	All (*n* = 6399)			Paramedic (*n* = 5023)			Physician (*n* = 695)			Specialist (*n* = 667)		
Variables	Non-RSI (*n* = 3236)	RSI (*n* = 3163)	*p*	Non-RSI (*n* = 2591)	RSI (*n* = 2432)	*p*	Non-RSI (*n* = 329)	RSI (*n* = 366)	*p*	Non-RSI (*n* = 307)	RSI (*n* = 360)	*p*
Age, y mean (SD)	65.2 (18.2)	62.2 (19.0)	<0.0001	65.9 (17.2)	62.8 (18.4)	<0.0001	62.7 (21.1)	59.8 (20.4)	0.07	61.9 (22.4)	60.9 (20.9)	0.53
Age, Median (range)	68 (0–102)	65 (1–100)	-	68 (0–99)	65 (2–100)	-	67 (0–94)	63 (4–94)	-	68 (0–94)	64 (1–98)	-
Male * (*n*, %)	1663 51.4%	1775 56.1%	0.0009	1352 52.2%	1357 55.8%	0.02	174 52.9%	194 53.0%	0.89	136 45.3%	223 61.9%	<0.0001

* 22 missing.

**Table 2:** Indications and trauma rates.

	All (*N* = 6399)			Paramedic (*N* = 5023)			Physician (*N* = 695)			Specialist (*N* = 667)		
Variables	Non-RSI (*n* = 3236)	RSI (*n* = 3163)	*p*	Non-RSI (*n* = 2591)	RSI (*n* = 2432)	*p*	Non-RSI (*n* = 329)	RSI (*n* = 366)	*p*	Non-RSI (*n* = 307)	RSI (*n* = 360)	*p*
Airway compromise	1065 (32.9%)	616 (19.5%)	<0.0001	864 (33.4%)	445 (18.3%)	<0.0001	111 (33.7%)	92 (25.1%)	0.0128	85 (27.7%)	77 (21.4%)	0.0587
Unconsciousness	2348 (72.6%)	2392 (75.6%)	0.005	1886 (72.8%)	1834 (75.4%)	0.0342	233 (70.8%)	280 (76.5%)	0.0889	223 (72.6%)	274 (76.1%)	0.3050
Ventilatory failure	1232 (38.1%)	872 (27.6%)	<0.0001	973 (37.6%)	682 (28.0%)	<0.0001	133 (40.4%)	97 (26.5%)	<0.0001	125 (40.7%)	91 (25.3%)	<0.0001
Agitation and head injury	67 (2.1%)	122 (3.9%)	<0.0001	48 (1.9%)	87 (3.6%)	0.0002	8 (2.4%)	22 (6.0%)	0.0204	11 (3.6%)	13 (3.6%)	0.9845
Others	99 (3.1%)	79 (2.5%)	0.18	68 (2.6%)	53 (2.2%)	0.3038	16 (4.9%)	15 (4.1%)	0.6258	15 (4.9%)	11 (3.1%)	0.2235
Trauma	313 (9.7%)	468 (14.8%)	<0.0001	238 (9.2%)	342 (14.1%)	<0.0001	36 (10.9%)	64 (17.5%)	0.01	39 (12.7%)	61 (16.9%)	0.13

**Table 3:** Results/complications.

	All (*N* = 6399)			Paramedic (*N* = 5023)			Physician (*N* = 695)			Specialist (*N* = 667)		
Variables	Non-RSI (*n* = 3236)	RSI (*n* = 3163)	*p*	Non-RSI (*n* = 2591)	RSI (*n* = 2432)	*p*	Non-RSI (*n* = 329)	RSI (*n* = 366)	*p*	Non-RSI (*n* = 307)	RSI (*n* = 360)	*p*
Successful ETI	3120 (96.4%)	3071 (97.1%)	0.13	2494 (96.3%)	2355 (96.8%)	0.2632	318 (96.7%)	357 (97.5%)	0.4862	299 (97.4%)	355 (98.6%)	0.2571
EtCO_2_ + *	2502 (77.3%)	3087 (97.6%)	<0.0001	1986 (76.7%)	2386 (98.1%)	<0.0001	262 (79.6%)	347 (94.8%)	<0.0001	249 (81.1%)	349 (96.9%)	<0.0001
First-attempt success rate	2422 (74.9%)	2706 (85.6%)	<0.0001	1908 (73.6%)	2078 (85.4%)	<0.0001	253 (76.9%)	310 (84.7%)	0.0089	257 (83.7%)	314 (87.2%)	0.1982
Hypoxia	249 (7.7%)	264 (8.3%)	0.34	198 (7.6%)	208 (8.6%)	0.2366	28 (8.5%)	29 (7.9%)	0.7782	22 (7.2%)	27 (7.5%)	0.8692
Hypotension	631 (19.5%)	421 (13.3%)	<0.0001	506 (19.5%)	319 (13.1%)	<0.0001	76 (23.1%)	50 (13.7%)	0.0013	48 (15.6%)	51 (14.2%)	0.5949
CA	107 (3.3%)	85 (2.7%)	0.15	82 (3.2%)	65 (2.7%)	0.3011	12 (3.7%)	13 (3.6%)	0.9462	13 (4.2%)	7 (1.9%)	0.0839
DASH-1A	1780 (55.0%)	2165 (68.5%)	<0.0001	1399 (54.0%)	1667 (68.5%)	<0.0001	181 (55.0%)	244 (66.7%)	0.0017	198 (64.5%)	251 (69.7%)	0.1514

* tube position confirmed by EtCO_2_ measurement.

**Table 4:** Subgroup analyses.

Comparison	Subgroup (s)	*n*	DASH-1A OR (95% CI)
RSI vs. non-RSI	Paramedic subgroup	5023	1.86 (1.65–2.08)
RSI vs. non-RSI	Physician subgroup	695	1.64 (1.20–2.22)
RSI vs. non-RSI	Specialist subgroup	667	1.27 (0.92–1.75)
RSI vs. non-RSI	Non-trauma patients	5618	1.75 (1.57–1.95)
RSI vs. non-RSI	Trauma patients	781	2.09 (1.55–2.80)
Trauma vs. non-trauma (within RSI group)	RSI group	3163	0.96 (0.78–1.19)
Trauma vs. non-trauma (within non-RSI group)	Non-RSI group	3236	0.81 (0.64–1.02)
RSI vs. non-RSI	No airway compromise	4718	1.8 (1.6–2.1)
RSI vs. non-RSI	Airway compromise	1681	1.4 (1.2–1.8)
RSI vs. non-RSI	No ventilatory failure	4295	1.9 (1.7–2.2)
RSI vs. non-RSI	Ventilatory failure	2104	1.4 (1.2–1.7)

## Data Availability

The original structured data presented in this study were retrained from patient documentation using a dedicated report form (RF) and are openly available in the Supplementary Materials.

## Supplementary Materials

The following supporting information can be downloaded at https://www.mdpi.com/article/10.3390/life15111725/s1. Figure S1: RSI algorithm; database: dataset.

## Abbreviations

The following abbreviations are used in this manuscript:

RSI	Rapid Sequence Intubation
ETI	Endotracheal Intubation
EMS	Emergency Medical Service
NAS	National Ambulance Service, Hungary
SOP	Standard Operating Procedure
SD	Standard Deviation
DASH-1A	Definitive Airway Sans Hypoxia and Hypotension on First Attempt
CA	Cardiac Arrest
HEMS	Helicopter Emergency Medical Service

## References

[1] Pepe P.E., Roppolo L.P., Fowler R.L. (2015). Prehospital endotracheal intubation: Elemental or detrimental?. Crit. Care.

[2] Crewdson K., Lockey D., Voelckel W., Temesvari P., Lossius H.M., EHAC Medical Working Group (2019). Best practice advice on pre-hospital emergency anaesthesia & advanced airway management. Scand. J. Trauma Resusc. Emerg. Med..

[3] Fevang E., Perkins Z., Lockey D., Jeppesen E., Lossius H.M. (2017). A systematic review and meta-analysis comparing mortality in pre-hospital tracheal intubation to emergency department intubation in trauma patients. Crit. Care.

[4] Denninghoff K.R., Nuno T., Pauls Q., Yeatts S.D., Silbergleit R., Palesch Y.Y., Merck L.H., Manley G.T., Wright D.W. (2017). Prehospital Intubation is Associated with Favorable Outcomes and Lower Mortality in ProTECT III. Prehospital Emerg. Care.

[5] Khosravan S., Alami A., Hamzei A., Borna J. (2015). Comparing the effectiveness of airway management devices in pre-hospital emergency care: A randomized clinical trial. Pak. J. Med. Sci..

[6] Bossers S.M., Schwarte L.A., Loer S.A., Twisk J.W., Boer C., Schober P. (2015). Experience in Prehospital Endotracheal Intubation Significantly Influences Mortality of Patients with Severe Traumatic Brain Injury: A Systematic Review and Meta-Analysis. PLoS ONE.

[7] Benoit J.L., Gerecht R.B., Steuerwald M.T., McMullan J.T. (2015). Endotracheal intubation versus supraglottic airway placement in out-of-hospital cardiac arrest: A meta-analysis. Resuscitation.

[8] Hasegawa K., Hiraide A., Chang Y., Brown D.F. (2013). Association of prehospital advanced airway management with neurologic outcome and survival in patients with out-of-hospital cardiac arrest. JAMA.

[9] Evans C.C., Brison R.J., Howes D., Stiell I.G., Pickett W. (2013). Prehospital non-drug assisted intubation for adult trauma patients with a Glasgow Coma Score less than 9. Emerg. Med. J..

[10] Lossius H.M., Roislien J., Lockey D.J. (2012). Patient safety in pre-hospital emergency tracheal intubation: A comprehensive meta-analysis of the intubation success rates of EMS providers. Crit. Care.

[11] Wang H.E., Balasubramani G.K., Cook L.J., Lave J.R., Yealy D.M. (2010). Out-of-hospital endotracheal intubation experience and patient outcomes. Ann. Emerg. Med..

[12] Bernard S.A., Nguyen V., Cameron P., Masci K., Fitzgerald M., Cooper D.J., Walker T., Std B.P., Myles P., Murray L. (2010). Prehospital rapid sequence intubation improves functional outcome for patients with severe traumatic brain injury: A randomized controlled trial. Ann. Surg..

[13] Tam R.K., Maloney J., Gaboury I., Verdon J.M., Trickett J., Leduc S.D., Poirier P. (2009). Review of endotracheal intubations by Ottawa advanced care paramedics in Canada. Prehospital Emerg. Care.

[14] Berlac P., Hyldmo P.K., Kongstad P., Kurola J., Nakstad A.R., Sandberg M. (2008). Pre-hospital airway management: Guidelines from a task force from the Scandinavian Society for Anaesthesiology and Intensive Care Medicine. Acta Anaesthesiol. Scand..

[15] Wang H.E., Yealy D.M. (2006). Out-of-hospital endotracheal intubation: Where are we?. Ann. Emerg. Med..

[16] Burton J.H. (2006). Out-of-hospital endotracheal intubation: Half empty or half full?. Ann. Emerg. Med..

[17] Seamon M.J., Doane S.M., Gaughan J.P., Kulp H., D’Andrea A.P., Pathak A.S., Santora T.A., Goldberg A.J., Wydro G.C. (2013). Prehospital interventions for penetrating trauma victims: A prospective comparison between Advanced Life Support and Basic Life Support. Injury.

[18] DeMasi S.C., Casey J.D., Semler M.W. (2025). Evidence-based Emergency Tracheal Intubation. Am. J. Respir. Crit. Care Med..

[19] Ben-Naoui I., Compère V., Clavier T., Besnier E. (2025). Practices of Rapid Sequence Induction for Prevention of Aspiration—An International Declarative Survey. J. Clin. Med..

[20] Jarvis J.L., Lyng J.W., Miller B.L., Perlmutter M.C., Abraham H., Sahni R. (2022). Prehospital Drug Assisted Airway Management: An NAEMSP Position Statement and Resource Document. Prehospital Emerg. Care.

[21] Apfelbaum J.L., Hagberg C.A., Connis R.T., Abdelmalak B.B., Agarkar M., Dutton R.P., Fiadjoe J.E., Greif R., Klock P.A., Mercier D. (2022). 2022 American Society of Anesthesiologists Practice Guidelines for Management of the Difficult Airway. Anesthesiology.

[22] Mosier J.M., Sakles J.C., Law J.A., Brown C.A., Brindley P.G. (2020). Tracheal Intubation in the Critically Ill. Where We Came from and Where We Should Go. Am. J. Respir. Crit. Care Med..

[23] Lauriks A., Missiaen M., Sabbe M. (2025). Prehospital intubation in patients with severe traumatic brain injury: A review. Eur. J. Emerg. Med..

[24] Fouche P.F., Stein C., Simpson P., Carlson J.N., Doi S.A. (2017). Nonphysician Out-of-Hospital Rapid Sequence Intubation Success and Adverse Events: A Systematic Review and Meta-Analysis. Ann. Emerg. Med..

[25] Crewdson K., Lockey D.J., Roislien J., Lossius H.M., Rehn M. (2017). The success of pre-hospital tracheal intubation by different pre-hospital providers: A systematic literature review and meta-analysis. Crit. Care.

[26] Peters J., van Wageningen B., Hendriks I., Eijk R., Edwards M., Hoogerwerf N., Biert J. (2015). First-pass intubation success rate during rapid sequence induction of prehospital anaesthesia by physicians versus paramedics. Eur. J. Emerg. Med..

[27] Heritage D., Griggs J., Barrett J., Clarke S., Carroll R., Lyon R., Bootland D. (2024). Helicopter emergency medical services demonstrate reduced time to emergency anaesthesia in an undifferentiated trauma population: A retrospective observational analysis across three major trauma networks. Scand. J. Trauma. Resusc. Emerg. Med..

[28] Phelps S. (2017). Intubation success rates of prehospital rapid sequence induction of anaesthesia by physicians versus paramedics. Eur. J. Emerg. Med..

[29] Lockey D.J., Crewdson K., Davies G., Jenkins B., Klein J., Laird C., Mahoney P.F., Nolan J., Pountney A., Shinde S. (2017). AAGBI: Safer pre-hospital anaesthesia 2017: Association of Anaesthetists of Great Britain and Ireland. Anaesthesia.

[30] Myers L.A., Gallet C.G., Kolb L.J., Lohse C.M., Russi C.S. (2016). Determinants of Success and Failure in Prehospital Endotracheal Intubation. West. J. Emerg. Med..

[31] McQueen C., Crombie N., Hulme J., Cormack S., Hussain N., Ludwig F., Wheaton S. (2015). Prehospital anaesthesia performed by physician/critical care paramedic teams in a major trauma network in the UK: A 12 month review of practice. Emerg. Med. J..

[32] Soti A., Temesvari P., Hetzman L., Eross A., Petroczy A. (2015). Implementing new advanced airway management standards in the Hungarian physician staffed Helicopter Emergency Medical Service. Scand. J. Trauma. Resusc. Emerg. Med..

[33] Lockey D.J., Crewdson K. (2018). Pre-hospital anaesthesia: No longer the ‘poor relative’ of high quality in-hospital emergency airway management. Br. J. Anaesth..

[34] Crewdson K., Rehn M., Lockey D. (2018). Airway management in pre-hospital critical care: A review of the evidence for a ‘top five’ research priority. Scand. J. Trauma. Resusc. Emerg. Med..

[35] Hayes-Bradley C., McCreery M., Delorenzo A., Bendall J., Lewis A., Bowles K.-A. (2024). Predictive and protective factors for failing first pass intubation in prehospital rapid sequence intubation: An aetiology and risk systematic review with meta-analysis. Br. J. Anaesth..

[36] Sorbello M., Paternò D.S., Zdravkovic I., La Via L. (2025). Pharmacological approach to rapid sequence induction/intubation: A contemporary perspective. Curr. Opin. Anaesthesiol..

[37] Collins J., O’Sullivan E.P. (2022). Rapid sequence induction and intubation. BJA Educ..

[38] Raatiniemi L., Lankimaki S., Martikainen M. (2013). Pre-hospital airway management by non-physicians in Northern Finland—A cross-sectional survey. Acta Anaesthesiol. Scand..

[39] Gellerfors M., Fevang E., Backman A., Kruger A., Mikkelsen S., Nurmi J., Rognas L., Sandstrom E., Skallsjo G., Svensen C. (2018). Pre-hospital advanced airway management by anaesthetist and nurse anaesthetist critical care teams: A prospective observational study of 2028 pre-hospital tracheal intubations. Br. J. Anaesth..

[40] Harris T., Lockey D. (2011). Success in physician prehospital rapid sequence intubation: What is the effect of base speciality and length of anaesthetic training?. Emerg. Med. J..

[41] (2010). UK HEMS RSI SOP. http://www.ukhems.co.uk/Rapid%20Sequence%20Intubation.Pdf.

[42] Grmec S. (2002). Comparison of three different methods to confirm tracheal tube placement in emergency intubation. Intensive Care Med..

[43] Knapp S., Kofler J., Stoiser B., Thalhammer F., Burgmann H., Posch M., Hofbauer R., Stanzel M., Frass M. (1999). The assessment of four different methods to verify tracheal tube placement in the critical care setting. Anesth. Analg..

[44] Silvestri S., Ralls G.A., Krauss B., Thundiyil J., Rothrock S.G., Senn A., Carter E., Falk J. (2005). The effectiveness of out-of-hospital use of continuous end-tidal carbon dioxide monitoring on the rate of unrecognized misplaced intubation within a regional emergency medical services system. Ann. Emerg. Med..

[45] Lockey D.J., Avery P., Harris T., Davies G.E., Lossius H.M. (2013). A prospective study of physician pre-hospital anaesthesia in trauma patients: Oesophageal intubation, gross airway contamination and the ‘quick look’ airway assessment. BMC Anesth..

[46] Donald M.J., Paterson B. (2006). End tidal carbon dioxide monitoring in prehospital and retrieval medicine: A review. Emerg. Med. J..

[47] Higgs A., McGrath B.A., Goddard C., Rangasami J., Suntharalingam G., Gale R., Cook T.M., Difficult Airway Society, Intensive Care Society, Faculty of Intensive Care Medicine (2018). Guidelines for the management of tracheal intubation in critically ill adults. Br. J. Anaesth..

[48] Hinckley B. (2012). DASH-1A. https://prehospitalmed.com/2012/05/15/dash-1a-emergency-airway-concepts-with-dr-bill-hinckley/.

[49] Powell E.K., Hinckley W.R., Stolz U., Golden A.J., Ventura A., McMullan J.T. (2019). Predictors of Definitive Airway Sans Hypoxia/Hypotension on First Attempt (DASH-1A) Success in Traumatically Injured Patients Undergoing Prehospital Intubation. Prehospital Emerg. Care.

[50] Reichert R.J., Gothard M., Gothard M.D., Schwartz H.P., Bigham M.T. (2018). Intubation Success in Critical Care Transport: A Multicenter Study. Prehospital Emerg. Care.

[51] Sunde G.A., Heltne J.K., Lockey D., Burns B., Sandberg M., Fredriksen K., Hufthammer K.O., Soti A., Lyon R., Jantti H. (2015). Airway management by physician-staffed Helicopter Emergency Medical Services—A prospective, multicentre, observational study of 2327 patients. Scand. J. Trauma. Resusc. Emerg. Med..

[52] Sunde G.A., Sandberg M., Lyon R., Fredriksen K., Burns B., Hufthammer K.O., Roislien J., Soti A., Jantti H., Lockey D. (2017). Hypoxia and hypotension in patients intubated by physician staffed helicopter emergency medical services—A prospective observational multi-centre study. BMC Emerg. Med..

[53] Delorenzo A., St Clair T., Andrew E., Bernard S., Smith K. (2018). Prehospital Rapid Sequence Intubation by Intensive Care Flight Paramedics. Prehospital Emerg. Care.

[54] George N.H., Cihla J.B., Guyette F.X., Ramgopal S., Martin-Gill C. (2025). Prehospital Endotracheal Intubation Success Rates for Critical Care Nurses Versus Paramedics. Prehospital Emerg. Care.

[55] Park L., Zeng I., Brainard A. (2017). Systematic review and meta-analysis of first-pass success rates in emergency department intubation: Creating a benchmark for emergency airway care. Emerg. Med. Australas..

[56] Elmer J., Brown F., Martin-Gill C., Guyette F.X. (2019). Prevalence and Predictors of Post-Intubation Hypotension in Prehospital Trauma Care. Prehospital Emerg. Care.

