# Supplementary material for: Standardized Rapid Sequence Intubation (RSI) Improves Effectiveness and Safety in Mixed Physician and Paramedic Hungarian EMS
Source: Life (Basel). 2025 Nov 7;15(11):1725. doi: 10.3390/life15111725 (PMC12653650; doi:10.3390/life15111725)
Supplement: Supplementary file 1 [file life-15-01725-s001.zip › Figures Suppl Dig Cont A.pptx]

## Slide 1
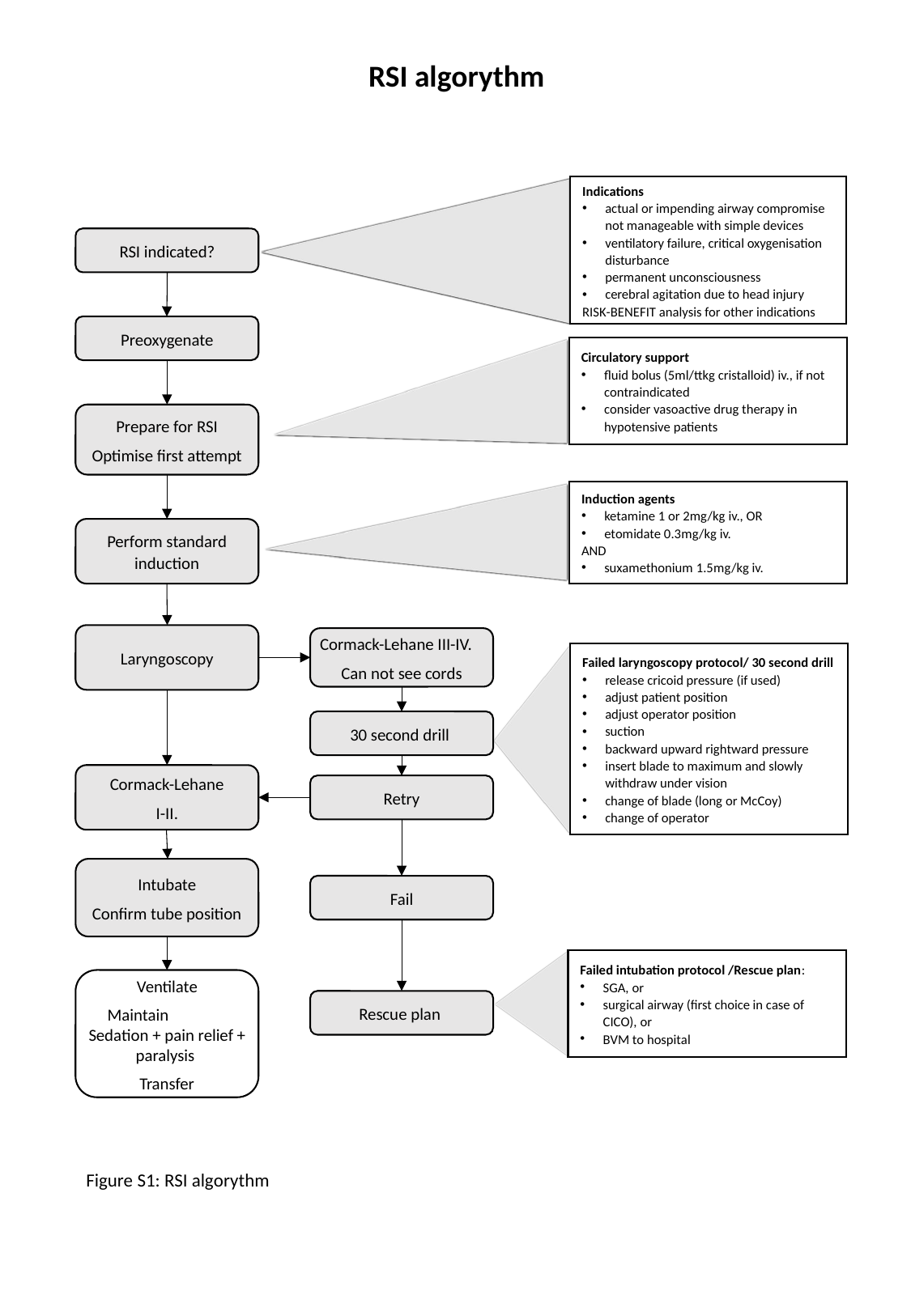

RSI algorythm
Indications
actual or impending airway compromise not manageable with simple devices
ventilatory failure, critical oxygenisation disturbance
permanent unconsciousness
cerebral agitation due to head injury
RISK-BENEFIT analysis for other indications
RSI indicated?
Preoxygenate
Circulatory support
fluid bolus (5ml/ttkg cristalloid) iv., if not contraindicated
consider vasoactive drug therapy in hypotensive patients
Prepare for RSI
Optimise first attempt
Induction agents
ketamine 1 or 2mg/kg iv., OR
etomidate 0.3mg/kg iv.
AND
suxamethonium 1.5mg/kg iv.
Perform standard induction
Laryngoscopy
Cormack-Lehane III-IV.
Can not see cords
Failed laryngoscopy protocol/ 30 second drill
release cricoid pressure (if used)
adjust patient position
adjust operator position
suction
backward upward rightward pressure
insert blade to maximum and slowly withdraw under vision
change of blade (long or McCoy)
change of operator
30 second drill
Cormack-Lehane
I-II.
Retry
Intubate
Confirm tube position
Fail
Failed intubation protocol /Rescue plan:
SGA, or
surgical airway (first choice in case of CICO), or
BVM to hospital
Ventilate
Maintain Sedation + pain relief + paralysis
Transfer
Rescue plan
Figure S1: RSI algorythm
